# Active smoking among people with diabetes mellitus or hypertension in Africa: a systematic review and meta-analysis

**DOI:** 10.1038/s41598-018-37858-z

**Published:** 2019-01-24

**Authors:** Jean Jacques Noubiap, Jobert Richie Nansseu, Francky Teddy Endomba, Anderson Ngouo, Jan René Nkeck, Ulrich Flore Nyaga, Arnaud D. Kaze, Jean Joel Bigna

**Affiliations:** 1 Department of Medicine, Groote Schuur Hospital and University of Cape Town, Cape Town, South Africa; 2 Department of Public Health, Faculty of Medicine and Biomedical Sciences, University of Yaoundé 1, Yaoundé, Cameroon; 3 Department for the Control of Disease, Epidemics and Pandemics, Ministry of Public Health, Yaoundé, Cameroon; 4 Department of Internal Medicine and Specialties, Faculty of Medicine and Biomedical Sciences, University of Yaoundé 1, Yaoundé, Cameroon; 5 Brigham and Women’s Hospital, Harvard Medical School, Boston, MA USA; 6 Department of Epidemiology and Public Health, Centre Pasteur of Cameroon, Yaoundé, Cameroon; 7 Faculty of Medicine, University of Paris Sud XI, Le Kremlin-Bicêtre, France

## Abstract

The objective was to summarize existing data on the prevalence of active tobacco smoking among patients with hypertension or diabetes mellitus in Africa. We searched PubMed, EMBASE, and AJOL to include studies published from January 01, 2000 to August 23, 2017 reporting on the prevalence of active smoking in individuals aged ≥15 years with hypertension or diabetes mellitus residing inside Africa. We used a random-effects meta-analysis model to pool studies. The pooled prevalence of active smoking among patients with hypertension or diabetes was 12.9% (95%CI: 10.6–15.3; 50 studies; 16,980 patients) and 12.9% (95%CI: 9.6–16.6; 42 studies; 18,564 patients), respectively. For both conditions, the prevalence of active smoking was higher in males than in females (p < 0.001), and in Northern compared to sub-Saharan Africa (p < 0.001). There was no difference between urban and rural settings, and between community-based and hospital-based studies, except for patients with diabetes for whom the prevalence was higher in hospital-based studies (p = 0.032). The prevalence of active smoking is high among patients with hypertension or diabetes mellitus in Africa, with the heaviest burden in Northern Africa. Interventions for smoking prevention or cessation should be implemented in these high risk populations, targeting particularly the males.

## Introduction

The burden of cardiovascular diseases (CVD) has dramatically risen in Africa over the past decade, and CVD and there is an epidemiological transition in which the burden of CVD is overtaking that of infectious diseases on the continent by 2030^1^. In sub-Saharan Africa (SSA) for instance, CVD were responsible for nearly 1 million deaths in 2013, representing 38.3% of non-communicable disease-related deaths and 11.3% of all-cause mortality^2^. This surge in the burden of CVD is driven by the increasing prevalence in cardiovascular risk factors^1^.

Hypertension, diabetes mellitus, hypercholesterolemia, obesity, and smoking are the five major modifiable traditional cardiovascular risk factors^3–5^. At least one of these five risk factors is present in 80% to 95% of individuals who experienced a fatal or non-fatal cardiovascular event^4,5^. The most recent data from the Global Burden of Disease study showed that hypertension, diabetes mellitus and smoking remain among the five leading factors contributing to the global burden of disease^6^. Much more, the interaction between these three risk factors is devastating. Indeed, all forms of smoking amplifies markedly the risk of all-cause, CVD and non-CVD morbidity and mortality in patients with hypertension and diabetes^7,8^.

Smoking cessation and prevention is therefore a crucial component in the management of hypertension and diabetes mellitus^7^. In Africa where hypertension and diabetes mellitus are highly prevalent, the magnitude of active smoking in patients with these conditions is not well known. We present here a systematic review and meta-analysis which estimates the prevalence of active smoking among patients with hypertension or diabetes in Africa.

## Methods

This review is reported in accordance with the Preferred Reporting Items for Systematic Reviews and Meta-Analyses (PRISMA) guidelines. The protocol was published in a peer-reviewed journal^9^, and is registered with PROSPERO (Registration number CRD42016052560). For this review, we used the same method as in previously published meta-analysis of prevalence studies^10–13^.

### Literature search

We searched PubMed, Excerpta Medica Database (EMBASE), and African Journals Online (AJOL) to identify all relevant articles published from January 01, 2000 to August 23, 2017 on the prevalence of active smoking in individuals with hypertension or diabetes mellitus in Africa. No language restriction was applied. The full search strategy was published in the study protocol^9^. The reference list of all relevant articles were screened to identify other potential data sources.

### Selection of studies for inclusion in the review

Cross-sectional and cohort studies reporting on the prevalence of active smoking in individuals aged more than 15 years with hypertension or diabetes mellitus residing in African continent or enough data to compute it were included. Hypertension had to be defined as the presence of systolic blood pressure ≥140 mmHg and/or diastolic blood pressure ≥90 mmHg or being on any antihypertensive treatment^14^. Diabetes mellitus was defined according to one of the following diagnostic criteria: being on any antidiabetic treatment, A1c haemoglobin ≥6.5%or fasting plasma glucose ≥126 mg/dL (7.0 mmol/L) or 2 hours plasma glucose ≥200 mg/dL (11.1 mmol/L) or random plasma glucose ≥200 mg/dL (11.1 mmol/L) in the presence of classic symptoms of hyperglycemia^15^. Active smoking was defined as current use of any tobacco product in either smoked or smokeless form^16^. We excluded studies conducted among populations of African origin residing outside Africa, studies on non-systemic hypertension (intracranial hypertension, pulmonary hypertension) or studies on gestational diabetes, letters, case series with small sample size (less than 50 participants), reviews, commentaries and editorials. For studies published in more than one paper, the most comprehensive one reporting the largest sample size was considered.

Titles and abstracts of articles retrieved from literature search were independently screened by two investigators (JJN and JJB), and the full-texts of those potentially eligible were obtained and further assessed for final inclusion. Disagreements were resolved through consensus.

### Assessment of methodological quality and reporting of data

Methodological quality of included studies was evaluated using the tool developed by Hoy and colleagues^17^. A score of 1 (yes) or 0 (no) was assigned for each item, and scores summed across items to generate an overall quality score that ranged from 0 to 10. Studies were then classified as having a low (>8), moderate (6–8), or high (≤5) risk of bias. Three investigators (UFN, AN and JRNkeck) independently assessed study methodological quality of a third of included studies for each of them, and all the assessments were independently reviewed by a fourth investigator (JJN) with disagreements being resolved through consensus.

### Data extraction and management

A preconceived and standardized Google online data extraction form was used to collect information on first author’s name, study country, African sub-region (Northern Africa vs sub-Saharan Africa), year of publication, study design (cross-sectional, cohort or case-control), setting (population-based vs hospital-based), area (rural vs urban), number of participants, mean or median age of the population, proportion of males, definition of smoking and the prevalence of active smoking. Three investigators (UF, AN and JRNkeck) extracted the data from individual studies, and all extracted data were crosschecked by a fourth investigator (JJN) with disagreements being resolved through consensus.

### Data synthesis and analysis

We performed statistical analysis with R version 3.5.1 (The R Foundation for statistical computing, Vienna, Austria). Meta-analyses were conducted with the package ‘*meta*’. Unadjusted prevalence was recalculated based on the information of crude number of cases and sample size provided by each individual study. Each prevalence was reported with its 95% confidence interval (95%CI). The variance of each included study was stabilized with the Freeman-Tukey double arcsine transformation before meta-analysis. This was done to keep the effect of studies with extremely small or extremely large prevalence estimates on the overall estimate to a minimum^18^. Random-effects analysis was used to pool data. Funnel plot was drawn to investigate any asymmetry. The formal Egger’s test was used to definitively identify publication bias if p value < 0.10^19^. Heterogeneity was evaluated by the χ² test on Cochrane’s Q statistic^20^. The I² statistic, used to quantify heterogeneity, estimated the percentage of total variation across studies due to true between-study differences rather than chance. The I² values greater than 60–70% indicated the presence of substantial heterogeneity^21^. We also used H statistics to quantify heterogeneity. Subgroup analyses were performed for the following subgroups: sex (male versus female), regions (northern versus sub-Sahara Africa), sub-regions (northern, southern, central, eastern, and western), areas (urban versus rural), and settings (population versus hospital-based studies). To test for an effect of study and participants’ characteristics (year of publication, proportion of males, regions, areas, setting, and sample size), we used univariable and multivariable meta-regression analyses. We applied a manual forward selection procedure to identify sources of heterogeneity independently associated with the variation of overall prevalence of active tobacco smoking. We included in multivariable meta-regression analysis, all variables associated (p value < 0.20) with the variation of prevalence in univariable analysis. For categorical variables with 3 or more categories, the global p value was considered for the inclusion in multivariable models. A 2-sided p value < 0.05 was considered statistically significant.

### Role of funding source

This study received no funding. All authors had full access to all study data and the corresponding author had final responsibility for the decision to submit the paper for publication.

## Results

### The review process and study characteristics

**Figure 1:**
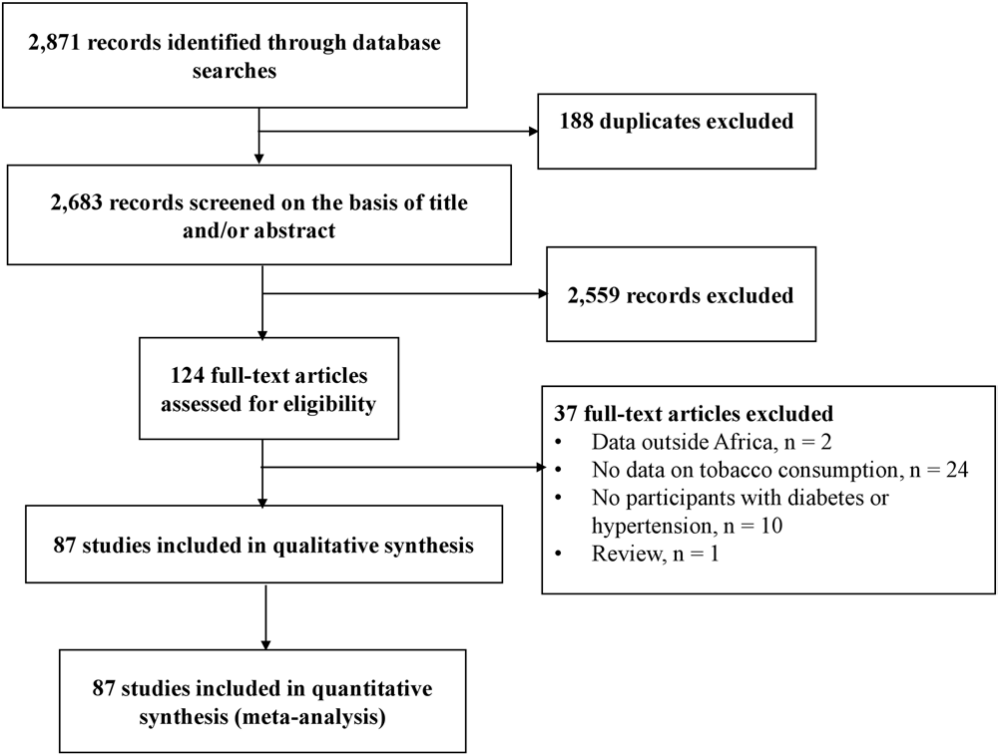
The review process.

Initially, 2,871 records were identified. After elimination of duplicates, 2,683 records remained. Titles and abstracts were screened and 2,559 irrelevant records were excluded. Of the remaining 124 papers (full texts) scrutinized for eligibility, 37 were excluded with reasons. Finally, 87 full texts were retained in the meta-analysis with 45 including data for hypertension only^22–66^, 37 for diabetes mellitus only^67–103^ and five including both conditions^104–108^ (Fig. 1).

Supplementary Table 1 (Appendix) summarizes characteristics of included studies. For hypertension, most studies were from western Africa, were multicentre, were conducted in urban areas, were population-based, and used prospective and consecutive sampling. For diabetes mellitus, most studies originated from Eastern and Northern Africa, were conducted in urban areas, in a single centre, were hospital-based, and used prospective and consecutive sampling. Forty-two (48.3%) studies had low, 38 (43.7%) studies had moderate and 7 (8.0%) studies had high risk of bias. Individual characteristics of each included study are shown in Supplementary Table 2 (Appendix).

### Prevalence of active smoking in hypertension

**Figure 2:**
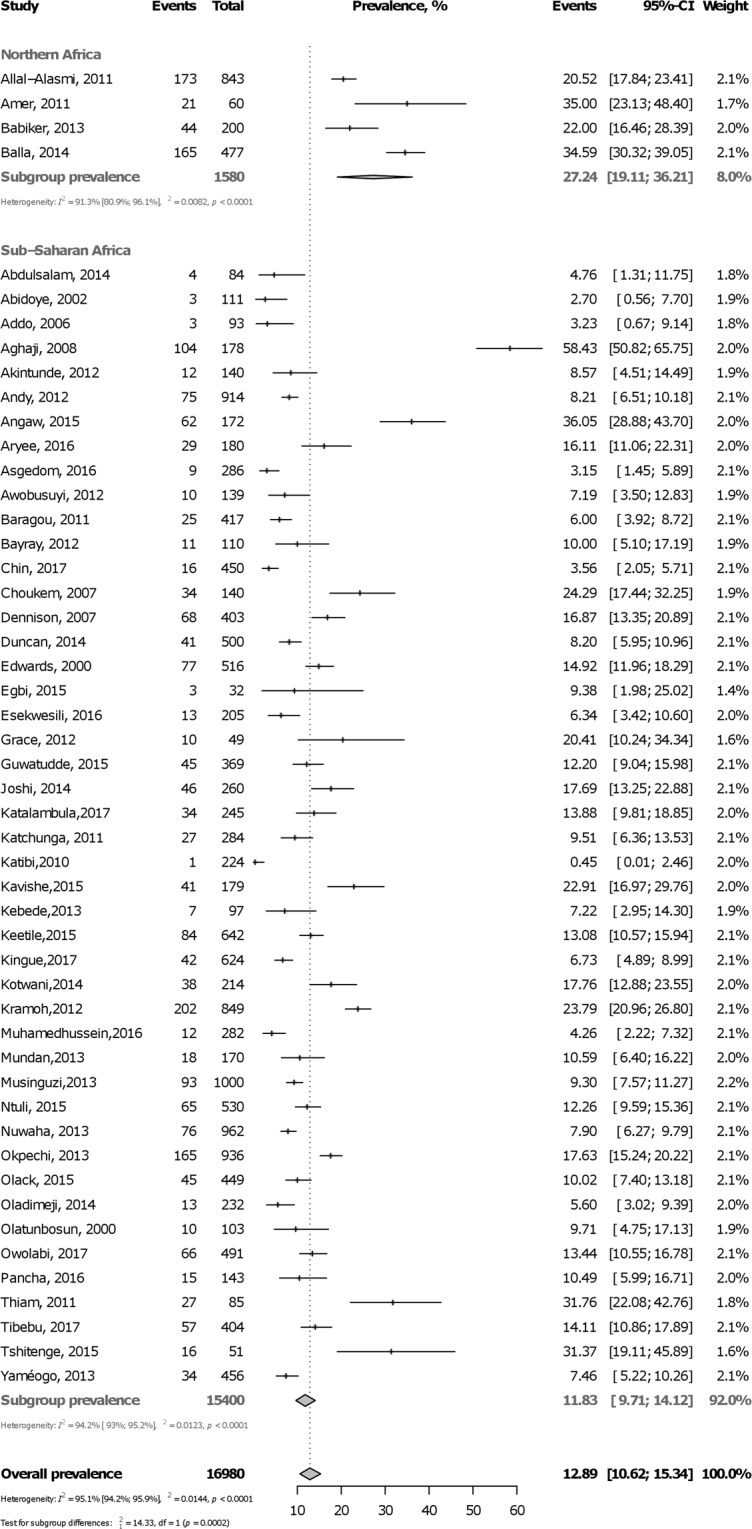
Forest plot of the meta-analysis prevalence of active smoking among people with hypertension in Africa.

In total, 16,980 participants were included from 18 countries. The prevalence varied widely from 0.5% to 58.4% both in Nigeria. Table 1 summarizes overall and subgroup statistics of the prevalence of active smoking in hypertension. The pooled overall prevalence of active smoking in hypertension was 12.9% (95%CI: 10.6–15.3; 50 studies) with substantial heterogeneity (Fig. 2). The prevalence was higher in males (27.6, 95%CI: 19.6–36.4) than in females (5.9, 95%CI: 4.1–8.0) (p < 0.0001) (Supplementary Figure 1, Appendix). The prevalence was higher in northern Africa (27.2%, 95%CI: 19.1–36.2) compared to sub-Saharan Africa (11.8%, 95%CI: 9.7–14.1) (p = 0.0002) (Fig. 2). This finding was confirmed for sub-regions analysis (Supplementary Figure 2, Appendix) and in meta-regression analysis (Supplementary Table 3, Appendix). There was no difference in smoking prevalence (p = 0.38 and 0.95, respectively) between rural (15.9%, 95%CI: 8.8–24.6) and urban dwellers (12.0%, 95%CI: 8.2–16.4) (Supplementary Figure 3, Appendix) and between hospital-based (12.8%, 95%CI: 9.0–17.0) and community-based studies (12.9%, 95%CI: 10.1–16.1) (Supplementary Figure 4, Appendix). There was no publication bias for overall (Supplementary Figure 5, Appendix) and subgroup analyses (Table 1).

**Table 1:** Summary statistics of the prevalence of active tobacco smoking in people with hypertension in Africa.

	Prevalence % (95% confidence interval)	N Studies	N Participants	H (95% confidence interval)	I² (95% confidence interval)	p heterogeneity	p Egger	p difference subgroups
Overall	12.9 (10.6–15.3)	50	16980	4.5 (4.1–4.9)	95.1 (94.2–95.9)	<0.0001	0.686	
By sex								
Male	27.6 (19.6–36.4)	8	1412	3.5 (2.7–4.5)	91.6 (85.9–95.0)	<0.0001	0.741	<0.0001
Female	5.9 (4.1–8.0)	7	2384	1.9 (1.3–2.8)	71.2 (37.5–86.8)	0.002	0.948	
By region								
Northern Africa	27.2 (19.1–36.2)	4	1580	3.4 (2.3–5.0)	91.3 (80.9–96.1)	<0.0001	0.620	0.0002
Sub-Saharan Africa	11.8 (9.7–14.1)	46	15400	4.2 (3.8–4.6)	94.2 (93.0–95.2)	<0.0001	0.604	
By sub-region								
Northern Africa	27.2 (19.1–36.2)	4	1580	3.4 (2.3–5.0)	91.3 (80.9–96.1)	<0.0001	0.620	0.005
Southern Africa	14.2 (11.0–17.6)	7	2666	2.3 (1.6–3.2)	80.2 (59.7–90.3)	<0.0001	0.109	
Central Africa	14.0 (6.6–23.6)	3	567	2.9 (6.6–23.6)	87.7 (65.5–95.6)	0.0003	0.538	
Eastern Africa	11.6 (8.6–15.1)	16	5796	3.8 (3.2–4.5)	93.0 (90.1–95.0)	<0.0001	0.195	
Western Africa	10.7 (6.4–15.8)	18	5378	5.3 (4.7–6.1)	96.5 (95.4–97.3)	<0.0001	0.489	
By area								
Urban	12.0 (8.2–16.4)	18	5237	4.4 (3.8–5.1)	94.9 (93.2–96.2)	<0.0001	0.447	0.388
Rural	15.9 (8.8–24.6)	11	3588	6.3 (5.5–7.4)	97.5 (96.6–98.2)	<0.0001	0.536	
Setting								
Population-based	13.0 (10.1–16.1)	32	11639	4.7 (4.3–5.2)	95.5 (94.5–96.3)	<0.0001	0.690	0.959
Hospital-based	12.8 (9.0–17.0)	18	5341	4.3 (3.7–4.9)	94.5 (92.6–95.9)	<0.0001	0.890

### Prevalence of active smoking in diabetes mellitus

**Figure 3:**
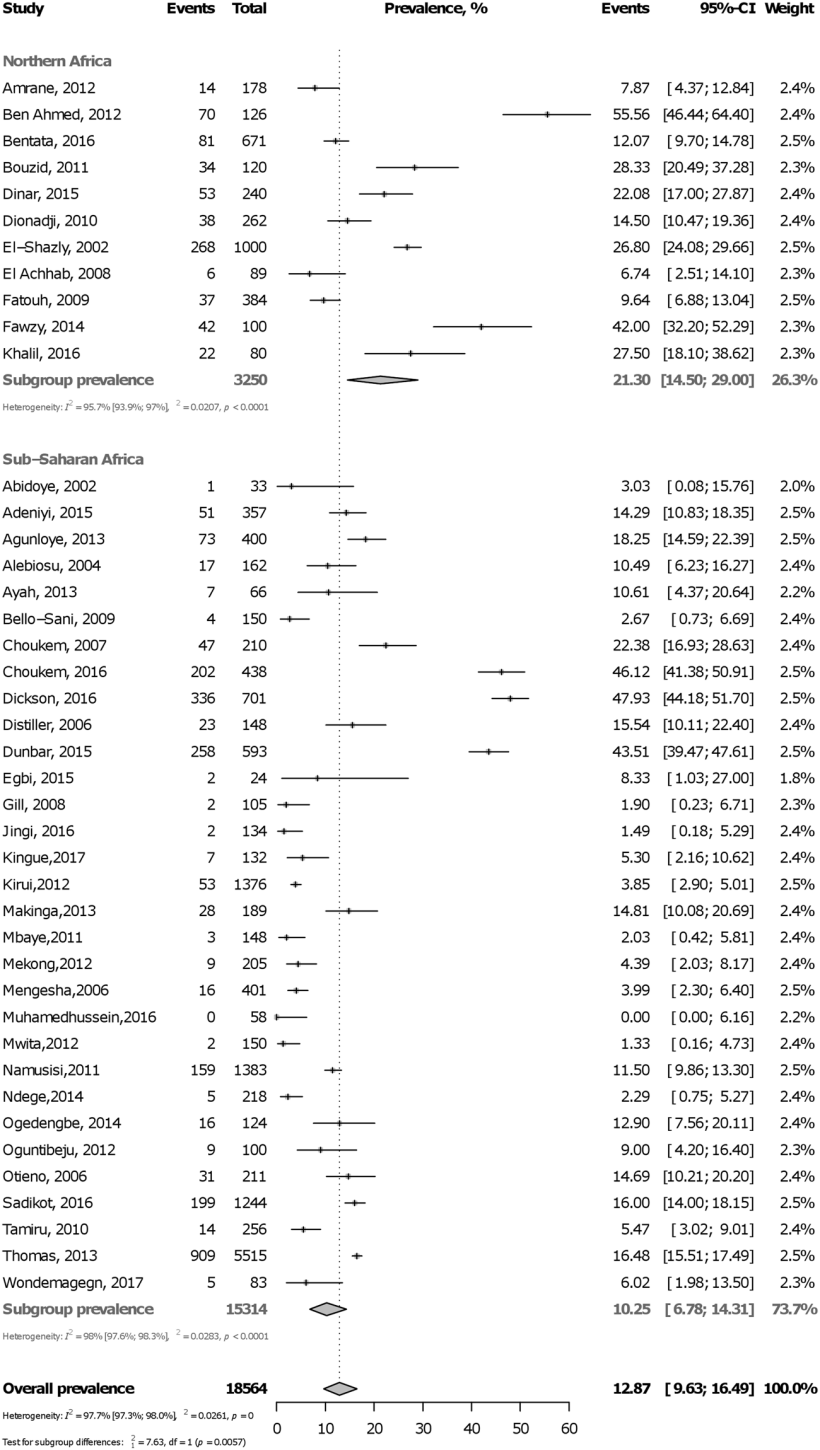
Forest plot of the meta-analysis prevalence of active smoking among people with diabetes mellitus in Africa.

In total, 18,564 participants were included from 18 countries. The prevalence varied widely, from 0.0% in Tanzania to 55.7% in Tunisia. Table 2 summarizes overall and subgroup statistics of the prevalence of active smoking in diabetes mellitus. The pooled overall prevalence of active smoking in diabetes mellitus was 12.9% (95%CI: 9.6–16.6; 42 studies) with substantial heterogeneity (Fig. 3). The prevalence was higher in males (18.6%, 95%CI: 9.7–29.6) than in females (2.1%, 95%CI: 0.0–6.5) (p = 0.0006) (Supplementary Figure 6, Appendix). The prevalence was higher in northern Africa (21.3%, 95%CI: 14.5–29.0) compared to sub-Saharan Africa (10.3%, 95%CI: 6.8–14.3) (p = 0.0002) (Fig. 3). This finding was confirmed for sub-regions analysis (Supplementary Figure 7, Appendix) and in meta-regression analysis (Supplementary Table 4, Appendix). There was no difference (p = 0.19) in the prevalence of active smoking between urban (11.7%, 95%CI: 7.7–16.3) and rural dwellers (3.8%, 95%CI: 0.0–16.0) (Supplementary Figure 8, Appendix). The prevalence of active smoking was higher (p = 0.032) in hospital-based (14.3%, 95%CI: 10.5–18.6) compared to community-based studies (6.6%, 95%CI: 2.5–12.3) (Supplementary Figure 9, Appendix). There was no publication bias for overall (Supplementary Figure 10, Appendix) analysis; however, there was publication bias for some subgroup analyses including females, central Africa, rural areas, and population-based studies (Table 2).

**Table 2:** Summary statistics of the prevalence of active tobacco smoking in people with diabetes mellitus in Africa.

	Prevalence % (95% confidence interval)	N Studies	N Participants	H (95% confidence interval)	I² (95% confidence interval)	p heterogeneity	p Egger	p difference subgroups
Overall	12.9 (9.6–16.6)	42	18564	6.7 (6.1–7.1)	97.7 (97.3–98.0)	<0.0001	0.372	
By sex								
Male	18.6 (9.7–29.6)	6	1130	4.0 (3.0–5.3)	93.6 (88.8–96.4)	<0.0001	0.752	0.0006
Female	2.1 (0.0–6.5)	6	1470	3.6 (2.7–4.9)	92.3 (86.0–95.8)	<0.0001	0.049	
By region								
Northern Africa	21.3 (14.5–29.0)	11	3250	4.8 (4.0–5.8)	95.7 (93.9–97.0)	<0.0001	0.671	0.006
Sub-Saharan Africa	10.3 (6.8–14.3)	31	15314	7.0 (6.5–7.6)	98.0 (97.6–98.3)	<0.0001	0.194	
By sub-region								
Northern Africa	21.3 (14.5–29.0)	11	3250	4.8 (4.0–5.8)	95.7 (93.9–97.0)	<0.0001	0.671	0.021
Southern Africa	16.8 (8.427.3)	6	7203	7.6 (6.3–9.2)	98.3 (97.5–98.8)	<0.0001	0.958	
Central Africa	15.0 (0.9–40.6)	4	987	9.0 (7.3–11.2)	98.8 (98.1–99.2)	<0.0001	0.065	
Western Africa	7.7 (3.5–13.3)	8	1141	2.8 (2.1–3.8)	87.6 (77.8–93.1)	<0.0001	0.232	
Eastern Africa	7.3 (1.8–15.8)	11	4607	8.5 (7.5–9.6)	98.6 (98.2–98.9)	<0.0001	0.612	
By area								
Urban	11.7 (7.7–16.3)	24	7010	5.4 (4.8–6.0)	96.5 (95.7–97.2)	<0.0001	0.570	0.191
Rural	3.8 (0.0–16.0)	3	520	4.1 (2.7–6.3)	94.0 (85.8–97.4)	<0.0001	0.090	
Setting								
Population-based	6.6 (2.5–12.3)	7	1989	3.3 (2.5–4.4)	91.0 (84.0–94.9)	<0.0001	0.029	0.032
Hospital-based	14.3 (10.5–18.6)	35	16575	7.0 (6.5–7.6)	98.0 (97.6–98.3)	<0.0001	0.669

## Discussion

This systematic review and meta-analysis aimed to estimate the prevalence of active tobacco smoking among patients with hypertension or diabetes mellitus living in Africa. We compiled data from about 20,000 patients for each condition, and obtained a pooled prevalence of 13%. There was a wide variation between countries, from 0.5% to 58.4% both in Nigeria for patients with hypertension, and from 0.0% in Tanzania to 55.7% in Tunisia for patients with diabetes mellitus, with substantial heterogeneity between studies. Additionally, we found for both conditions that the prevalence of active smoking was higher in males than in females and in Northern Africa than in sub-Saharan Africa. However, there was no difference in prevalence estimates between urban and rural settings, and between community-based and hospital-based studies, except for patients with diabetes mellitus for whom the prevalence of active smoking was higher in hospital-based compared to community-based studies.

The prevalence of 13% found in this review concurs roughly with other reports from hypertensive or diabetes populations residing outside Africa^109–111^. Likewise, our estimates align with what has been reported by the World Health Organization (WHO) for the prevalence of smoking among the African general population, around 12%^16^. Similarly, the wide variation of prevalence estimates observed between studies or countries was previously reported^16,112^. For instance, in a meta-analysis compiling data from 13 African countries mostly from Eastern, Western and Southern Africa, it was found that the prevalence of active tobacco smoking in the general population varied immensely, from 1.8 to 25.8%^112^. Notwithstanding, the prevalence of active tobacco smoking in Africa seems lower than in European or American countries^16,112,113^, though all forms of tobacco consumption need to be taken into account.

The higher prevalence of smoking in men compared to women is unsurprising and corroborates previous reports^16,112^. Indeed, the global prevalence of smoking is about five times higher in men (37%) than in women (7%). In Africa specifically, it is 22% of males in comparison to only 2% of females who smoke^16^. Although reasons for this huge discrepancy between men and women’s attitude towards smoking remains mostly unexplored in Africa, it was hypothesized that there might be an influence of the culture or societal behaviour which discourages women from smoking^114^. On the other hand, the absence of difference between rural and urban settings corroborates previous observations regarding the prevalence of smoking in the Sub-Saharan African general population^112^.

Moreover, our prevalence estimates of active smoking were more than twice higher in Northern Africa than in sub-Saharan Africa both for patients with hypertension (27.2 vs. 11.8%; p = 0.0002) or diabetes mellitus (21.3 vs. 10.3%; p = 0.0002). This similar prevalence in hypertensive and diabetes populations suggests that tobacco burden might be lower in the general population in sub-Saharan Africa compared to Northern Africa. In fact, the global status report on tobacco clearly shows a lower prevalence of smoking in low-income countries including most sub-Saharan African countries, as compared to middle-income countries, in which Northern African countries are classified^16^. This might be explained by the fact that cigarette is more affordable for populations with higher socioeconomic status. Furthermore, Northern African countries might be culturally more prone to smoke.

Accordingly, special attention should be given to Northern Africa when monitoring the policies and interventions to reduce tobacco use on the continent. Considering the current previsions which announce an exponential increment in the prevalence of tobacco in Africa by 22% by 2030^115^, it is likely that the prevalence of smoking in patients with hypertension or diabetes mellitus will also increase sharply. Despite these projections and up till now, tobacco control has received very low priority in Africa^115^. Indeed, Africa is still very far behind full implementation of the WHO Framework Convention on Tobacco Control guidelines^116^, particularly when it comes to protection from exposure to tobacco smoke, packaging and labelling of tobacco products, and tobacco advertising, promotion and sponsorship^117^. Most importantly, raising taxes on tobacco products which is the best cost-effective strategy to reduce the burden of tobacco consumption is weakly and sparsely implemented in Africa^16,117,118^.

Hence, it is high time African countries start adopting and implementing or reinforcing tobacco control strategies to reduce the current and/or future tobacco burden in the continent^16,119,120^. This will contribute substantially in preventing people from starting to smoke. On the other hand and singularly, context-specific interventions for smoking cessation should be implemented, especially in hypertensive and diabetes populations, considering the devastating interaction between smoking, hypertension and/or diabetes, resulting in a sharp increase in all-cause and cardiovascular morbidity and mortality^6,7^. Indeed, smoking cessation is associated with many important improvements in health and quality of life and is pivotal in cardiovascular disease prevention^121,122^. Several smoking cessation interventions including pharmacological treatment, physical exercise, individual and telephone have been shown to be efficacious^123,124^. Furthermore, concerns have been raised that some smoking cessation therapies such as nicotine replacement therapy, bupropion or varenicline may raise the risk of major cardiovascular disease events associated within the quitting period. However, it has been shown that these therapies do not increase the risk of cardiovascular disease^123,125^. Patients should be continuously educated, and care givers trained and well-equipped to provide adequate support to their patients for smoking prevention and cessation, including pharmacological and behavioural therapies.

However, our findings should be interpreted in the context of some drawbacks. For instance and common to the majority of meta-analyses of this type, we found a substantial heterogeneity between studies; but we undertook sub-group and meta-regression analyses which contributed significantly in identifying the major sources of variability. Moreover, African sub-regions were disproportionally represented and a high number of studies were hospital-based or used consecutive sampling, which may have led to an overestimation of prevalence estimates in individual studies or may have hindered the translatability of our results to the entire African continent. Despite these limitations and to the very best of our knowledge, this is the first systematic review and meta-analysis which gives a clear and comprehensive estimation of the burden of active smoking in people with hypertension and/or diabetes mellitus residing in Africa. We used rigorous methodological procedures and robust statistical analyses to generate our estimates. Additionally, most studies that were included had a low risk of bias in their methodological quality.

## Conclusion

This first systematic review and meta-analysis on the prevalence of active tobacco smoking among patients with hypertension or diabetes mellitus in Africa figured out a high burden of smoking in these populations. Accordingly, specific and effective interventions should be initiated or reinforced in these patients with either or both conditions, to prevent them from smoking or help them to be delivered from tobacco addiction. Special attention should be deserved to men and those living in Northern Africa.

## Supplementary information

Supplementary Information

## Data Availability

All data generated or analyzed during this study are included in this published article and its supplementary information files.
