# Supplementary material for: Active smoking among people with diabetes mellitus or hypertension in Africa: a systematic review and meta-analysis
Source: Sci Rep. 2019 Jan 24;9:588. doi: 10.1038/s41598-018-37858-z (PMC6345945; doi:10.1038/s41598-018-37858-z)

Active smoking among people with diabetes mellitus or hypertension in Africa: a systematic review and meta-analysis

Jean Jacques Noubiap, Jobert Richie Nansseu, Francky Teddy Endomba, Anderson Ngouo, Jan René Nkeck, Ulrich Flore Nyaga, Arnaud D. Kaze, Jean Joel Bigna

APPENDIX

Supplementary Table 1 : Characteristics of included studies 2
Supplementary Table 2 : Individual characteristics of included studies 3
Supplementary Table 3 : Univariate meta-regression analysis for factors associated with the variation of the prevalence of current tobacco smoking in people with hypertension in Africa 7
Supplementary Table 4: Univariate meta-regression analysis for factors associated with the variation of the prevalence of current tobacco smoking in people with diabetes in Africa 7

Supplementary Figure 1 : Prevalence of active smoking in people with hypertension in Africa according to sex 8
Supplementary Figure 2 : Prevalence of active smoking in people with hypertension in Africa according to sub-regions 9
Supplementary Figure 3 : Prevalence of active smoking in people with hypertension in Africa according to area 10
Supplementary Figure 4 : Prevalence of active smoking in people with hypertension in Africa according to area 11
Supplementary Figure 5 : Funnel plot for publication bias in the prevalence of active smoking in people with hypertension in Africa 12
Supplementary Figure 6 : Prevalence of active smoking in people with diabetes mellitus in Africa according to sex 13
Supplementary Figure 7 : Prevalence of active smoking in people with diabetes mellitus in Africa according to sub-regions 14
Supplementary Figure 8 : Prevalence of active smoking in people with diabetes mellitus in Africa according to area 15
Supplementary Figure 9 : Prevalence of active smoking in people with diabetes mellitus in Africa according to setting 16
Supplementary Figure 10 : Funnel plot for publication bias in the prevalence of active smoking in people with hypertension in Africa 17

Supplementary Table 1 : ***Characteristics of included studies***

**Characteristics**	**Hypertension, N = 50**	**Diabetes, N = 42**
Year of publication	2000-2017	2002-2017
Period, range	1996-2016 (n = 35)	2000-2016 (n = 27)
Mean/median age, range (year)	33.0-73.1 (n = 35)	33.4-68.0 (n = 35)
% of males	22-100 (n = 47)	24-100 (n = 39)
Regions		
- Sub-Saharan Africa	46	31
- Northern Africa	4	11
Sub-regions		
- Central Africa	3	4
- Eastern Africa	16	11
- Northern Africa	4	11
- Southern Africa	7	6
- Western Africa	18	8
- Multiregional	2	2
Number of centres		
- Single centre	27	32
- Multicentre	19	10
- Unclear	4	0
Area		
- Urban	18	24
- Rural	11	3
- Both	9	3
- Unclear	12	12
Setting		
- Population	32	7
- Hospital	18	35
Timing of data collection		
- Prospective	38	30
- Retrospective	5	9
- Unclear	7	3
Sampling		
- Consecutive	20	26
- Random	19	10
- Systematic	4	3
- Stratified consecutive	1	0
- Unclear	1	3

*Supplementary Table 2 : **Individual characteristics of included studies***

Study	Country	Design	Number of center	Area	Setting	Timing of data collection	Period	Sampling	Age, years	Limit age, years	%Male	Disease	Sample size, Hypertension	Sample size, diabetes mellitus	Risk of bias
Abdulsalam, 2014	Nigeria	Cross-sectional	Multi-center	Rural	Population	Prospective	2013-2014	Consecutive	36.4	18-87	45.2	Hypertension	84		Low
Abidoye, 2002	Nigeria	Cross-sectional	Single-center	Urban	Population	Prospective	2002	Random	NR	20-59	NR	Hypertension, Diabetes	111	33	Low
Addo, 2006	Ghana	Cross-sectional	Multi-center	Rural	Population	Prospective	2006	Random	42.4	18-99	30.0	Hypertension	93		Low
Adeniyi, 2015	South Africa	Cross-sectional	Single-center	Rural	Population	Retrospective	2013	NR	NR	30-	29.7	Diabetes		357	Low
Aghaji, 2008	Nigeria	Cross-sectional	Single-center	Rural	Population	Prospective	2006	Systematic	35	18-	59.6	Hypertension	178		Low
Agunloye, 2013	Nigeria	Cross-sectional	Single-center	Urban	Hospital	Prospective	2012	Systematic	53.3	15-82	46.6	Diabetes		400	Low
Akintunde, 2012	Nigeria	Cross-sectional	Single-center	Urban	Hospital	Prospective	NR	Consecutive	55.9	18-	47.1	Hypertension	140		Low
Alebiosu, 2004	Nigeria	Cross-sectional	Single-center	Urban	Hospital	Prospective	2000-2001	Consecutive	53.4	36-70	50.0	Diabetes		162	Low
Allal-Alasmi, 2011	Tunisia	Cross-sectional	Single-center	Urban	Population	Prospective	2004-2005	Random	NR	35-70	45.0	Hypertension	843		Low
Amer, 2011	Egypt	Case-control	Single-center	Urban, Rural	Hospital	Retrospective	NR	Systematic	NR	60-	50.0	Hypertension	60		Low
Amrane, 2012	Algeria	Case-control	Single-center	Urban	Hospital	Retrospective	2007-2008	Random	NR	40-	51.1	Diabetes		178	Low
Andy, 2012	Nigeria	Cross-sectional	Multi-center	Rural	Population	Prospective	NR	Consecutive	34.3	15-	41.6	Hypertension	914		Low
Angaw, 2015	Ethiopia	Cross-sectional	Single-center	Urban	Population	Prospective	2014	Consecutive	39	18-	44.4	Hypertension	172		Low
Aryee, 2016	Ghana	Cross-sectional	Single-center	Urban	Hospital	Retrospective	2012-2013	Random	NR	22-87	NR	Hypertension	180		Low
Asgedom, 2016	Ethiopia	Cross-sectional	Single-center	Urban	Hospital	Prospective	2015	Random	54.8	26-94	53.8	Hypertension	286		Low
Awobusuyi, 2012	Nigeria	Cross-sectional	Single-center	Urban	Population	Prospective	2006	Random	45	NR	42.8	Hypertension	139		Low
Ayah, 2013	Kenya	Cross-sectional	Single-center	Urban	Population	Prospective	NR	Random	33.4	18-	50.9	Diabetes		66	Low
Babiker, 2013	Sudan	Cross-sectional	Single-center	Urban	Hospital	Prospective	NR	Random	NR	20-	46.0	Hypertension	200		Low
Balla, 2014	Sudan	Cross-sectional	Multi-center	Rural	Population	Prospective	2012	Random	46.7	18-	39.3	Hypertension	477		Low
Baragou, 2011	Togo	Cross-sectional	Single-center	NR	Population	Prospective	2009	NR	NR	18-	NR	Hypertension	417		High
Bayray, 2012	Ethiopia	Case-control	Multi-center	Urban	Population	Prospective	2010-2011	Random	60.3	40-	49.0	Hypertension	110		Low
Bello-Sani, 2009	Nigeria	Cross-sectional	Single-center	Urban	Hospital	Prospective	NR	Consecutive	50.5	30-70	49.0	Diabetes		150	Low
Ben Ahmed, 2012	Tunisia	Case-control	Single-center	Urban	Hospital	Retrospective	NR	Random	61.4	NR	73.0	Diabetes		126	Low
Bentata, 2016	Morocco	Baseline data of a Cohort study	Single-center	Urban	Hospital	Prospective	2009	Consecutive	65	NR	NR	Diabetes		671	Low

Study	Country	Design	Number of center	Area	Setting	Timing of data collection	Period	Sampling	Age, years	Limit age, years	%Male	Disease	Sample size, Hypertension	Sample size, diabetes mellitus	Risk of bias
Bouzid, 2011	Tunisia	Cross-sectional	Single-center	Urban	Hospital	Prospective	NR	Consecutive	51.4	30-	59.0	Diabetes		120	Low
Chin, 2017	Uganda	Case-control	Multi-center	Urban	Population	Prospective	2014	Consecutive	NR	18-	61.5	Hypertension	450		Moderate
Choukem, 2007	Cameroon	Cross-sectional	Single-center	Urban	Hospital	Prospective	NR	Consecutive	56.6	15-86	50.0	Hypertension, Diabetes	140	210	Low
Choukem, 2016	Cameroon	Cross-sectional	Single-center	Urban	Hospital	Prospective	NR	Consecutive	56.3	20-85	56.0	Diabetes		438	Moderate
Dennison, 2007	South Africa	Cross-sectional	Multi-center	NR	Hospital	Prospective	2003-2004	Consecutive	52	35-65	45.4	Hypertension	403		Moderate
Dickson, 2016	Uganda	Cross-sectional	Single-center	NR	Hospital	Prospective	2014	Consecutive	NR	20-	NR	Diabetes		701	Moderate
Dinar, 2015	Morocco	Cross-sectional	Single-center	NR	Hospital	Prospective	2011-2012	Consecutive	49	20-65	40.4	Diabetes		240	Moderate
Dionadji, 2010	Algeria	Cross-sectional	Single-center	NR	Hospital	Prospective	2004-2005	Consecutive	59	NR	46.18	Diabetes		262	Moderate
Distiller, 2006	South Africa	Cross-sectional	Single-center	Urban	Hospital	Prospective	NR	Consecutive	48	19-76	51.35	Diabetes		148	Moderate
Dunbar, 2015	South Africa	Cross-sectional	Multi-center	NR	Hospital	Retrospective	2009-2011	Consecutive	62.2	NR	50.1	Diabetes		593	Moderate
Duncan, 2014	South Africa	Cross-sectional	Multi-center	Rural	Hospital	Retrospective	2012	Consecutive	58	23-98	22.0	Hypertension	500		Low
Edwards, 2000	Tanzania	Cross-sectional	Multi-center	Urban, Rural	Population	Prospective	1996-1997	Consecutive	NR	NR	72.51	Hypertension	516		Low
Egbi, 2015	Nigeria	Cross-sectional	Single-center	Urban	Hospital	Prospective	NR	Consecutive	52.9	16-89	37.6	Hypertension, Diabetes	32	24	Low
El Achhab, 2008	Morocco	Case-control	Multi-center	NR	Hospital	Prospective	2004-2006	Random	NR	40-	100.0	Diabetes		89	Moderate
El-Shazly, 2002	Egypt	Case-control	Multi-center	NR	Hospital	Prospective	2000-2001	Random	NR	NR	50.9	Diabetes		1000	Low
Esekwesili, 2016	Nigeria	Cross-sectional	Multi-center	NR	Population	Prospective	NR	Random	NR	17-79	47.81	Hypertension	205		Low
Fatouh, 2009	Egypt	Cross-sectional	Multi-center	NR	Hospital	Retrospective	2009	Random	55.2	17-	24.0	Diabetes		384	High
Fawzy, 2014	Egypt	Cross-sectional	Single-center	NR	Hospital	Prospective	2011-2012	Random	43.8	14-67	43.0	Diabetes		100	Moderate
Gill, 2008	Ethiopia	Baseline data of a Cohort study	Single-center	Rural	Hospital	Prospective	NR	Consecutive	41	16-77	70.0	Diabetes		105	Moderate
Grace, 2012	South Africa	Cross-sectional	Multi-center	NR	Population	Prospective	NR	Random	41.7	26-58	100.0	Hypertension	49		Moderate
Guwatudde, 2015	Uganda, South Africa, Tanzania, Nigeria	Baseline data of a Cohort study	Multi-center	Urban, Rural	Population	Retrospective	2011-2012	Random	NR	18-	35.4	Hypertension	369		Low
Jingi, 2016	Cameroon	Cross-sectional	Single-center	Urban	Hospital	Retrospective	NR	Consecutive	59.3	44-84	61.3	Diabetes		134	Low
Joshi, 2014	Kenya	Cross-sectional	Single-center	Rural	Population	Prospective	NR	Consecutive	33.4	18-90	50.9	Hypertension	260		Low
Katalambula,2017	Tanzania	Cross-sectional	Single-center	Urban	Population	Prospective	2016	Consecutive	40.7	25-64	42.4	Hypertension	245		Moderate
Katchunga, 2011	DR Congo	Cross-sectional	Single-center	Urban, Rural	Population	Prospective	NR	Random	NR	NR	36.5	Hypertension	284		Low
Katibi,2010	Nigeria	Cross-sectional	Single-center	NR	Hospital	Prospective	2002	Consecutive	57.7	35-82	44.6	Hypertension	224		Moderate
Kavishe,2015	Tanzania, Uganda	Cross-sectional	Multi-center	Urban, Rural	Population	Prospective	2012-2013	Random	33	NR	48.0	Hypertension	179		Low

Study	Country	Design	Number of center	Area	Setting	Timing of data collection	Period	Sampling	Age, years	Limit age, years	%Male	Disease	Sample size, Hypertension	Sample size, diabetes mellitus	Risk of bias
Kebede,2013	Ethiopia	Cross-sectional	Single-center	Urban	Hospital	Prospective	2012	Consecutive	42.3	15-81	42.0	Hypertension	97		Low
Keetile,2015	Botswana	Cross-sectional	Single-center	Urban, Rural	Population	Prospective	2007	Random	NR	25-64	32.1	Hypertension	642		Low
Khalil, 2016	Egypt	Case-control	Single-center	Urban	Hospital	Prospective	NR	Consecutive	54.6	NR	68.75	Diabetes		80	Moderate
Kingue,2017	Cameroon, Nigeria, DR of Congo, Madagascar	Cross-sectional	Multi-center	Urban	Hospital	Prospective	2011-2013	Consecutive	52.6	NR	42.6	Hypertension, Diabetes	624	132	Low
Kirui,2012	Kenya	Cross-sectional	Multi-center	Urban, Rural	Hospital	Retrospective	2007-2011	Consecutive	53.5	NR	45.0	Diabetes		1376	High
Kotwani,2014	Uganda	Cross-sectional	Single-center	Rural	Population	Prospective	2012	Consecutive	49.5	NR	52.8	Hypertension	214		Moderate
Kramoh,2012	Ivory Coast	Cross-sectional	Single-center	Urban	Hospital	Retrospective	2000-2009	Consecutive	73.1	65-98	41.0	Hypertension	849		Moderate
Makinga,2013	Lesotho	Cross-sectional	Multi-center	Urban	Hospital	Prospective	2004-2005	Consecutive	54.7	NR	24.5	Diabetes		189	Moderate
Mbaye,2011	Senegal	Cross-sectional	Single-center	Urban	Population	Prospective	2010	Random	43.4	15-96	31.0	Diabetes		148	Moderate
Mekong,2012	Cameroon	Cross-sectional	Single-center	Urban	Hospital	Prospective	2009-2010	Consecutive	57	29-85	43.6	Diabetes		205	Low
Mengesha,2006	Botswana	Cross-sectional	Multi-center	Urban	Hospital	Prospective	2003-2004	Consecutive	53.8	NR	28.4	Diabetes		401	Moderate
Muhamedhussein,2016	Tanzania	Cross-sectional	Single-center	Rural	Population	Prospective	2011	Consecutive	44.5	41-50	49.1	Hypertension, Diabetes	282	58	Moderate
Mundan,2013	Kenya	Case-control	Single-center	Urban	Hospital	Prospective	NR	Consecutive	45.1	NR	95.0	Hypertension	170		Moderate
Musinguzi,2013	Uganda	Cross-sectional	Multi-center	Urban, Rural	Population	Prospective	2012	Systematic	36.2	NR	35.5	Hypertension	1000		Moderate
Mwita,2012	Tanzania	Cross-sectional	Single-center	Urban	Hospital	Prospective	2010	Consecutive	51.6	NR	38.0	Diabetes		150	Moderate
Namusisi,2011	Uganda	Cross-sectional	Single-center	Urban	Hospital	Retrospective	2005-2010	Consecutive	39.6	NR	39.0	Diabetes		1383	Moderate
Ndege,2014	Kenya	Cross-sectional	Single-center	Urban	Hospital	Prospective	2011-2012	Systematic	56.6	NR	44.0	Diabetes		218	Moderate
Ntuli, 2015	South Africa	Cross-sectional	NR	Rural	Population	Prospective	2011-2012	NR	44.2	15-98	37.0	Hypertension	530		Moderate
Nuwaha, 2013	Uganda	Cross-sectional	Single-center	NR	Population	NR	NR	NR	36.5	NR	35.7	Hypertension	962		Moderate
Ogedengbe, 2014	Nigeria	Cross-sectional	Single-center	NR	Hospital	NR	NR	NR	58.6	30-	40.3	Diabetes		124	High
Oguntibeju, 2012	Nigeria	Baseline data of a Cohort study	Multi-center	NR	Hospital	NR	NR	NR	64.75	40-89	31.0	Diabetes		100	High
Okpechi, 2013	Nigeria	Cross-sectional	NR	Urban, Rural	Population	NR	2011-2012	NR	41.7	18-	47.9	Hypertension	936		Moderate
Olack, 2015	Kenya	Cross-sectional	Multi-center	Urban	Population	Prospective	2013	Random	46.7	35-64	42.0	Hypertension	449		Moderate
Oladimeji, 2014	Nigeria	Cross-sectional	Multi-center	NR	Population	NR	NR	Random	43	20-	62.2	Hypertension	232		Moderate
Olatunbosun, 2000	Nigeria	Cross-sectional	NR	NR	Population	NR	NR	Random	40.02	NR	58.0	Hypertension	103		Moderate
Otieno, 2006	Kenya	Cross-sectional	Single-center	NR	Hospital	Prospective	NR	Consecutive	NR	NR	42.7	Diabetes		211	Low
Owolabi, 2017	South Africa	Cross-sectional	Multi-center	NR	Hospital	Prospective	2016	NR	NR	18-	32.5	Hypertension	491		Moderate

Study	Country	Design	Number of center	Area	Setting	Timing of data collection	Period	Sampling	Age, years	Limit age, years	%Male	Disease	Sample size, Hypertension	Sample size, diabetes mellitus	Risk of bias
Pancha, 2016	Cameroon	Cross-sectional	NR	NR	Population	Prospective	2014	Stratified consecutive	36	18-93	48.6	Hypertension	143		Moderate
Sadikot, 2016	Benin, Congo, South Africa	Cross-sectional	Multi-center	Urban	Population	Prospective	NR	Consecutive	68	NR	63.0	Diabetes		1244	Low
Tamiru, 2010	Ethiopia	Cross-sectional	Single-center	Urban, Rural	Hospital	NR	2007	Random	45.3	18-	62.1	Diabetes		256	Moderate
Thiam, 2011	Senegal	Cross-sectional	Single-center	NR	Hospital	NR	NR	NR	57	NR	64.7	Hypertension	85		Low
Thomas, 2013	South Africa	Baseline data of a Cohort study	Single-center	NR	Hospital	Retrospective	2001-2010	Consecutive	50.8	NR	63.4	Diabetes		5515	High
Tibebu, 2017	Ethiopia	Cross-sectional	Multi-center	NR	Hospital	Prospective	2015	Random	54	18-	52.0	Hypertension	404		Moderate
Tshitenge, 2015	Botswana	Cross-sectional	Single-center	Rural	Population	NR	2008	Systematic	NR	20-82	37.0	Hypertension	51		High
Wondemagegn, 2017	Ethiopia	Cross-sectional	Single-center	Urban, Rural	Population	Prospective	2016	Systematic	49	25-	59.0	Diabetes		83	Moderate
Yaméogo, 2013	Burkina Faso	Cross-sectional	Single-center	Urban, Rural	Hospital	NR	2010-2011	Consecutive	NR	NR	43.2	Hypertension	456		Moderate

NR: Not reported

Supplementary Table 3 : ***Univariate meta-regression analysis for factors associated with the variation of the prevalence of current tobacco smoking in people with hypertension in Africa***

	**Coefficient**	**P value**
Year of publication	-0.0019 (-0.0107 ; 0.0069)	0.668
Age	-0.0014 (-0.0064 ; 0.0035)	0.577
% of males	0.0254 (-0.0895 ; 0.1402)	0.665
Region (Northern Africa)*		
- Sub-Saharan Africa	-0.1976 (-0.3160 ; -0.0793)	0.001
Area (Rural)		0.546
- Urban	-0.0545 (-0.1560 ; 0.0471)	
- Urban and rural	-0.0173 (-0.1355 ; 0.1010)	
Setting (Hospital)		
- Population	0.0019 (-0.0718 ; 0.0757)	0.959
Sample size	-0.0222 (-0.0648 ; 0.0204)	0.307

Residual heterogeneity I² = 94.1%, *explained heterogeneity = 16.6%

Supplementary Table 4: ***Univariate meta-regression analysis for factors associated with the variation of the prevalence of current tobacco smoking in people with diabetes in Africa***

	**Univariate model**		**Multivariate model**	
	Coefficient	P value	Coefficient	P value
Year of publication	0.0071 (-0.0058 ; 0.0200)	0.283		
Age	0.0064 (-0.0008 ; 0.0135)	0.080		
% of males	0.0022 (-0.0012 ; 0.0056)	0.197		
Region (Northern Africa)*				
- Sub-Saharan Africa	-0.1515 (-0.2677 ; -0.0352)	0.011	-0.1515 (-0.2677 ; -0.0352)	0.011
Area (Rural)		0.183		
- Urban	0.1441 (-0.0485 ; 0.3367)			
- Urban and rural	0.0212 (-0.2333 ; 0.2756)			
Setting (Hospital)				
- Population	0.1250 (-0.2662 ; 0.0162)	0.083		
Sample size	0.0422 (-0.0103 ; 0.0947)	0.115		

Residual heterogeneity I² = 97.7%, *total explained heterogeneity = 3.1%

Supplementary Figure 1 : Prevalence of active smoking in people with hypertension in Africa according to sex

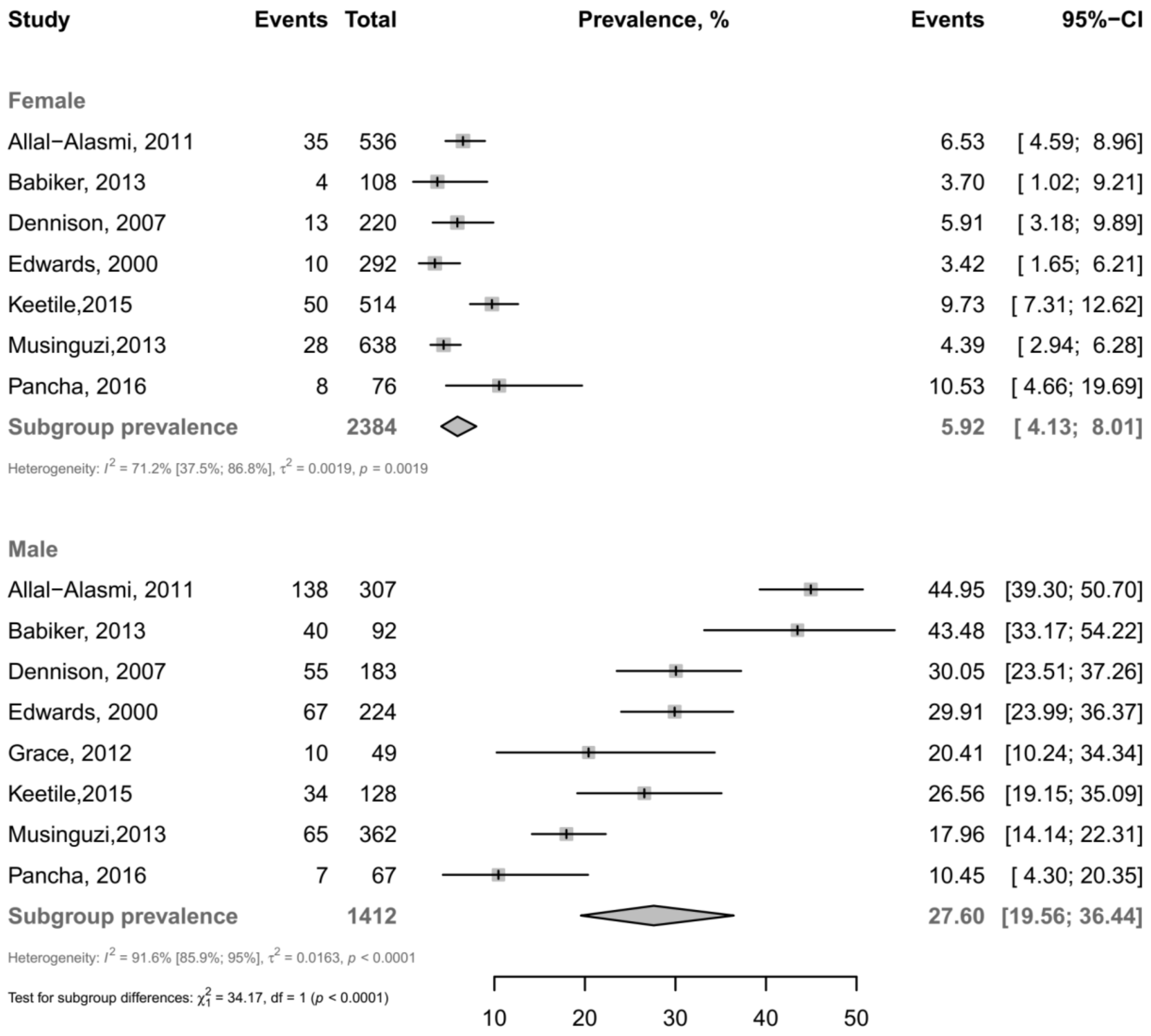

Supplementary Figure 2 : Prevalence of active smoking in people with hypertension in Africa according to sub-regions

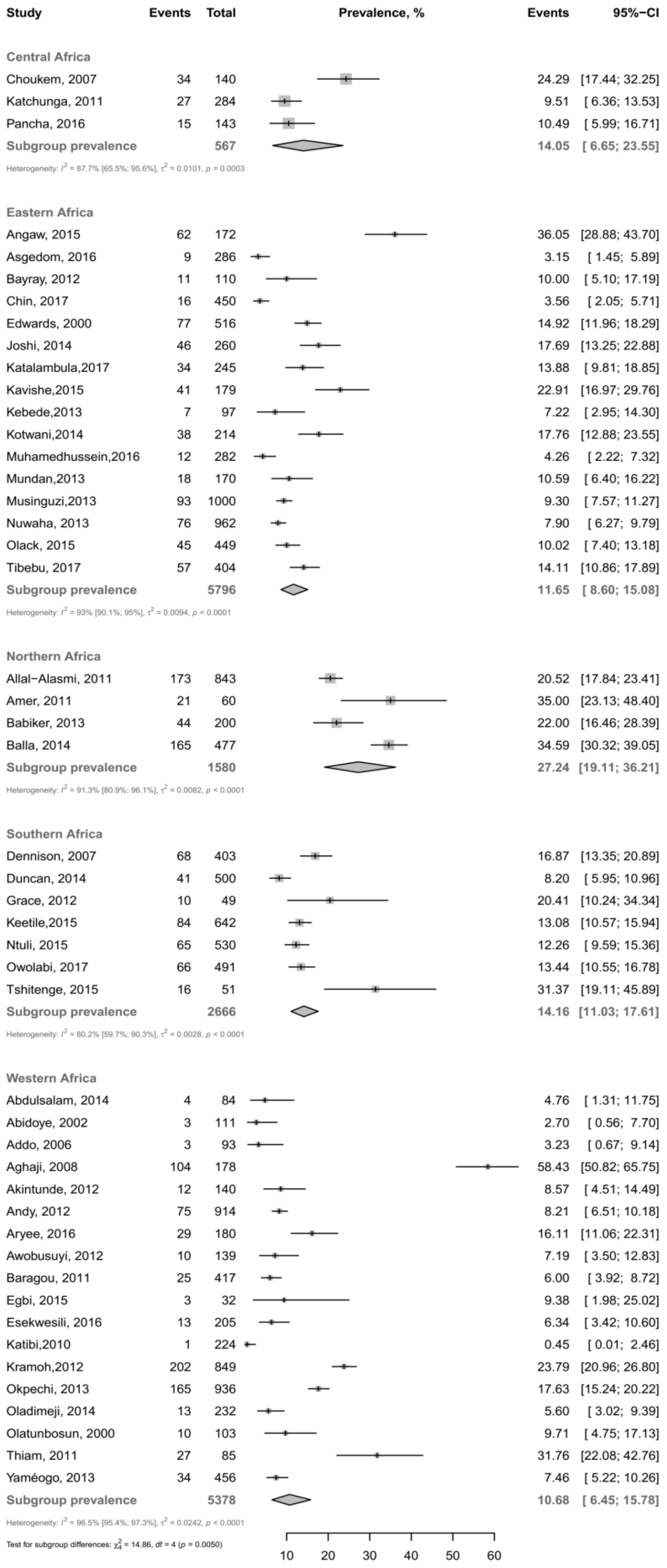

Supplementary Figure 3 : Prevalence of active smoking in people with hypertension in Africa according to area

Study	Events	Total	Events	95%−CI
**Rural**				
Abdulsalam, 2014	4	84	4.76	[1.31; 11.75]
Addo, 2006	3	93	3.23	[0.67; 9.14]
Aghaji, 2008	104	178	58.43	[50.82; 65.75]
Andy, 2012	75	914	8.21	[6.51; 10.18]
Balla, 2014	165	477	34.59	[30.32; 39.05]
Duncan, 2014	41	500	8.20	[5.95; 10.96]
Joshi, 2014	46	260	17.69	[13.25; 22.88]
Kotwani,2014	38	214	17.76	[12.88; 23.55]
Muhamedhussein,2016	12	282	4.26	[2.22; 7.32]
Ntuli, 2015	65	530	12.26	[9.59; 15.36]
Tshitenge, 2015	16	51	31.37	[19.11; 45.89]
**Subgroup prevalence**		**3583**	**15.90**	**[8.77; 24.60]**
Heterogeneity: I^2 = 97.5% [96.6%; 98.2%], τ^2 = 0.0319, p < 0.0001				
**Urban**				
Abidoye, 2002	3	111	2.70	[0.56; 7.70]
Akintunde, 2012	12	140	8.57	[4.51; 14.49]
Allal−Alasmi, 2011	173	843	20.52	[17.84; 23.41]
Angaw, 2015	62	172	36.05	[28.88; 43.70]
Aryee, 2016	29	180	16.11	[11.06; 22.31]
Asgedom, 2016	9	286	3.15	[1.45; 5.89]
Awobusuyi, 2012	10	139	7.19	[3.50; 12.83]
Babiker, 2013	44	200	22.00	[16.46; 28.39]
Bayray, 2012	11	110	10.00	[5.10; 17.19]
Chin, 2017	16	450	3.56	[2.05; 5.71]
Choukem, 2007	34	140	24.29	[17.44; 32.25]
Egbi, 2015	3	32	9.38	[1.98; 25.02]
Katalambula,2017	34	245	13.88	[9.81; 18.85]
Kebede,2013	7	97	7.22	[2.95; 14.30]
Kingue,2017	42	624	6.73	[4.89; 8.99]
Kramoh,2012	202	849	23.79	[20.96; 26.80]
Mundan,2013	18	170	10.59	[6.40; 16.22]
Olack, 2015	45	449	10.02	[7.40; 13.18]
**Subgroup prevalence**		**5237**	**12.03**	**[8.21; 16.44]**
Heterogeneity: I^2 = 94.9% [93.2%; 96.2%], τ^2 = 0.0168, p < 0.0001				

Test for subgroup differences: χ^2_1 = 0.74, df = 1 (p = 0.3881)

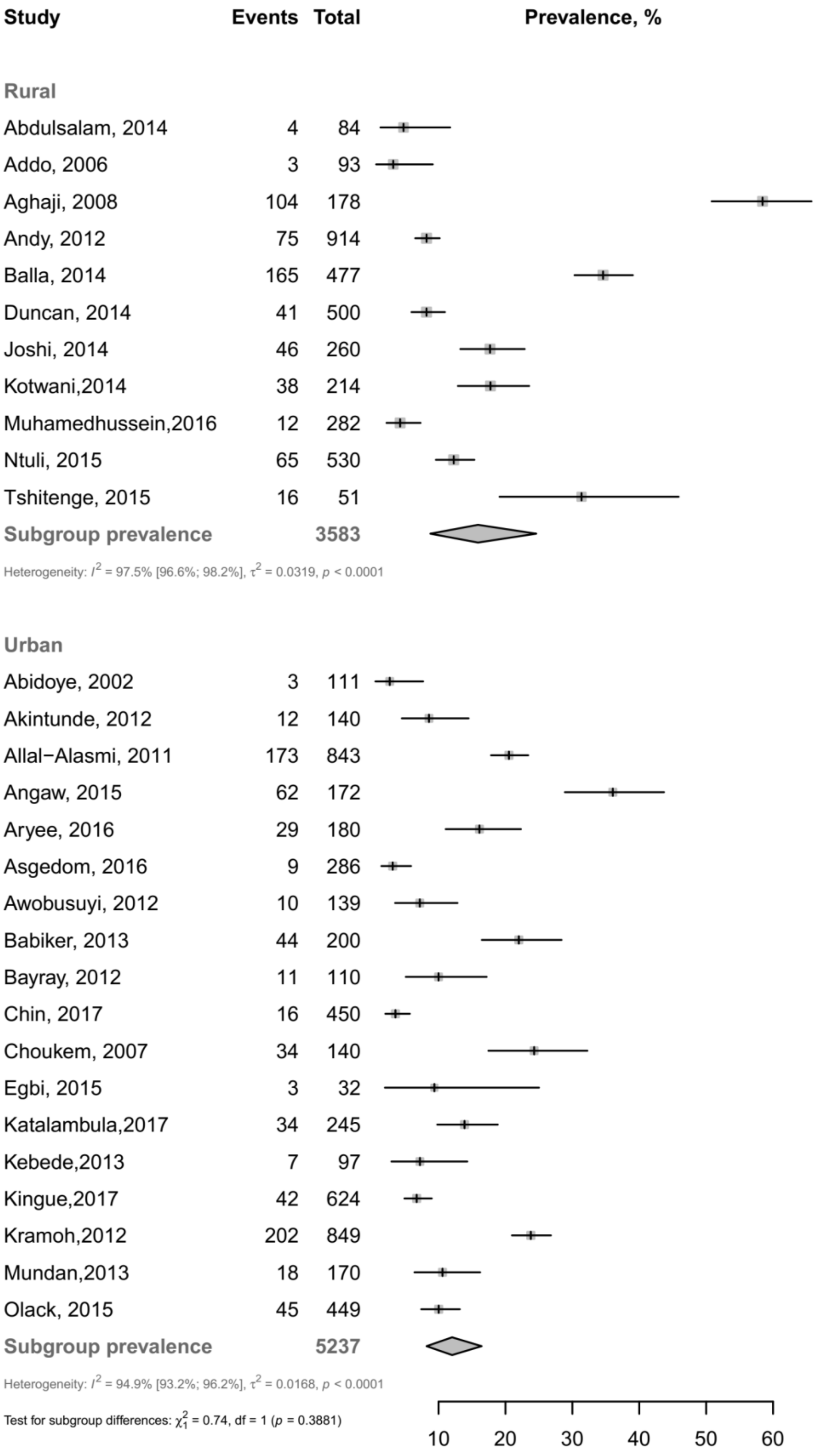

Supplementary Figure 4 : Prevalence of active smoking in people with hypertension in Africa according to area

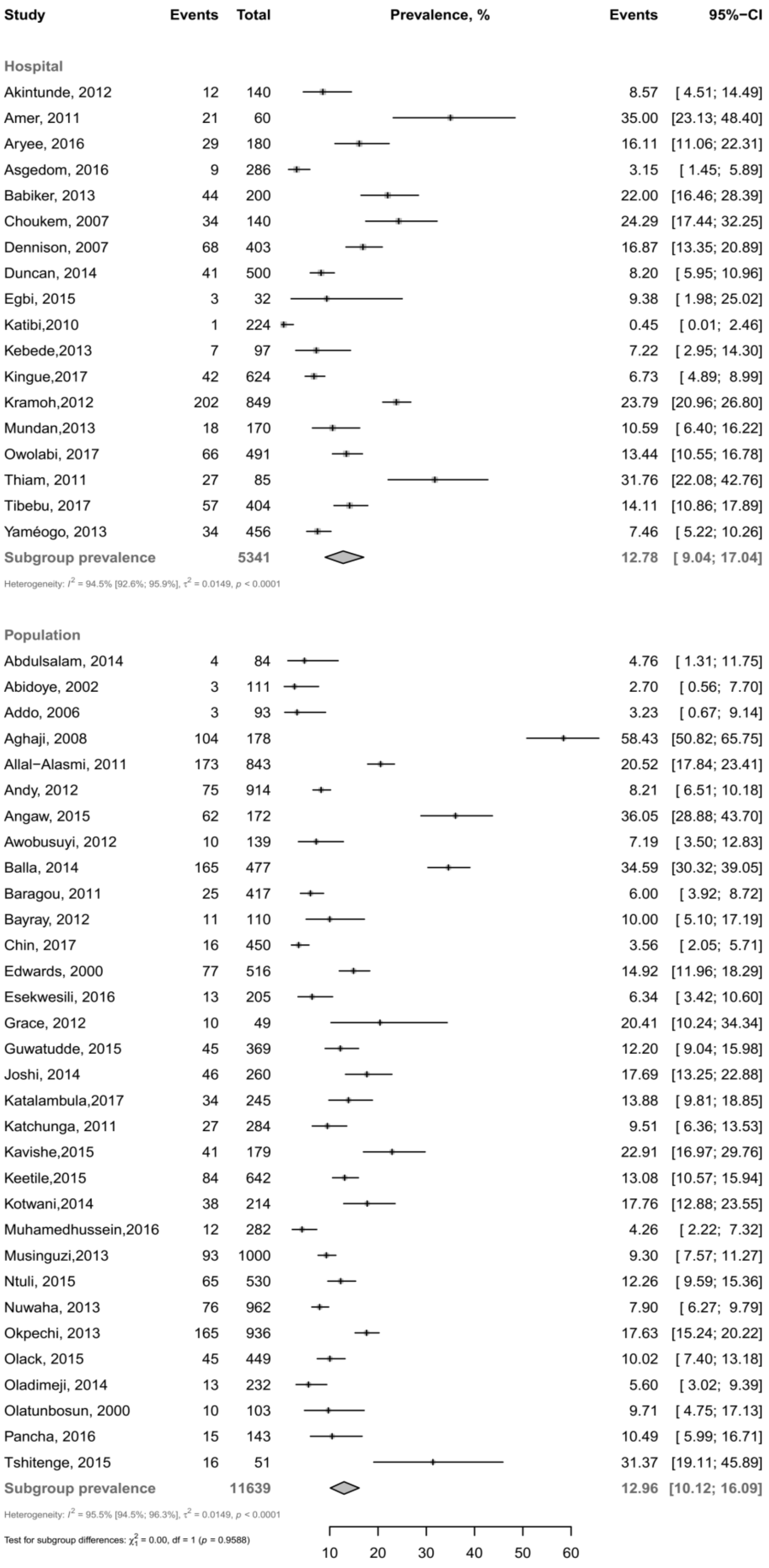

Supplementary Figure 5 : Funnel plot for publication bias in the prevalence of active smoking in people with hypertension in Africa

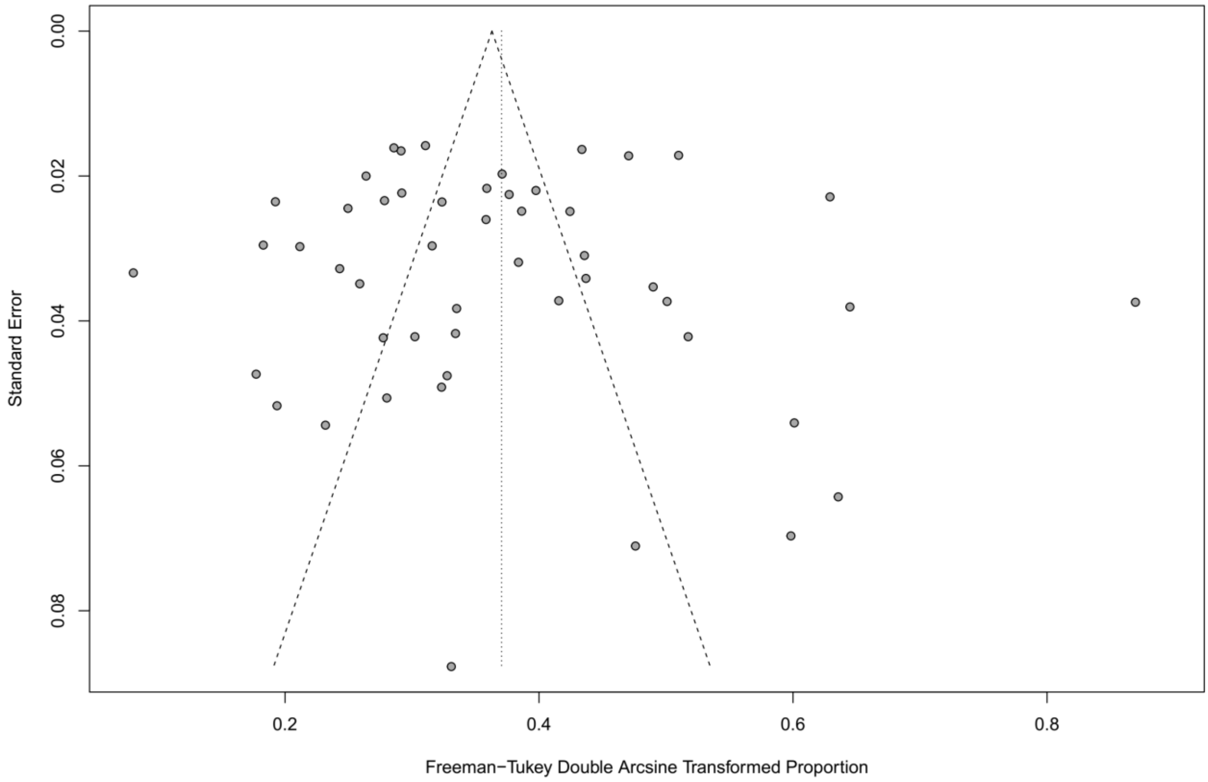

Supplementary Figure 6 : Prevalence of active smoking in people with diabetes mellitus in Africa according to sex

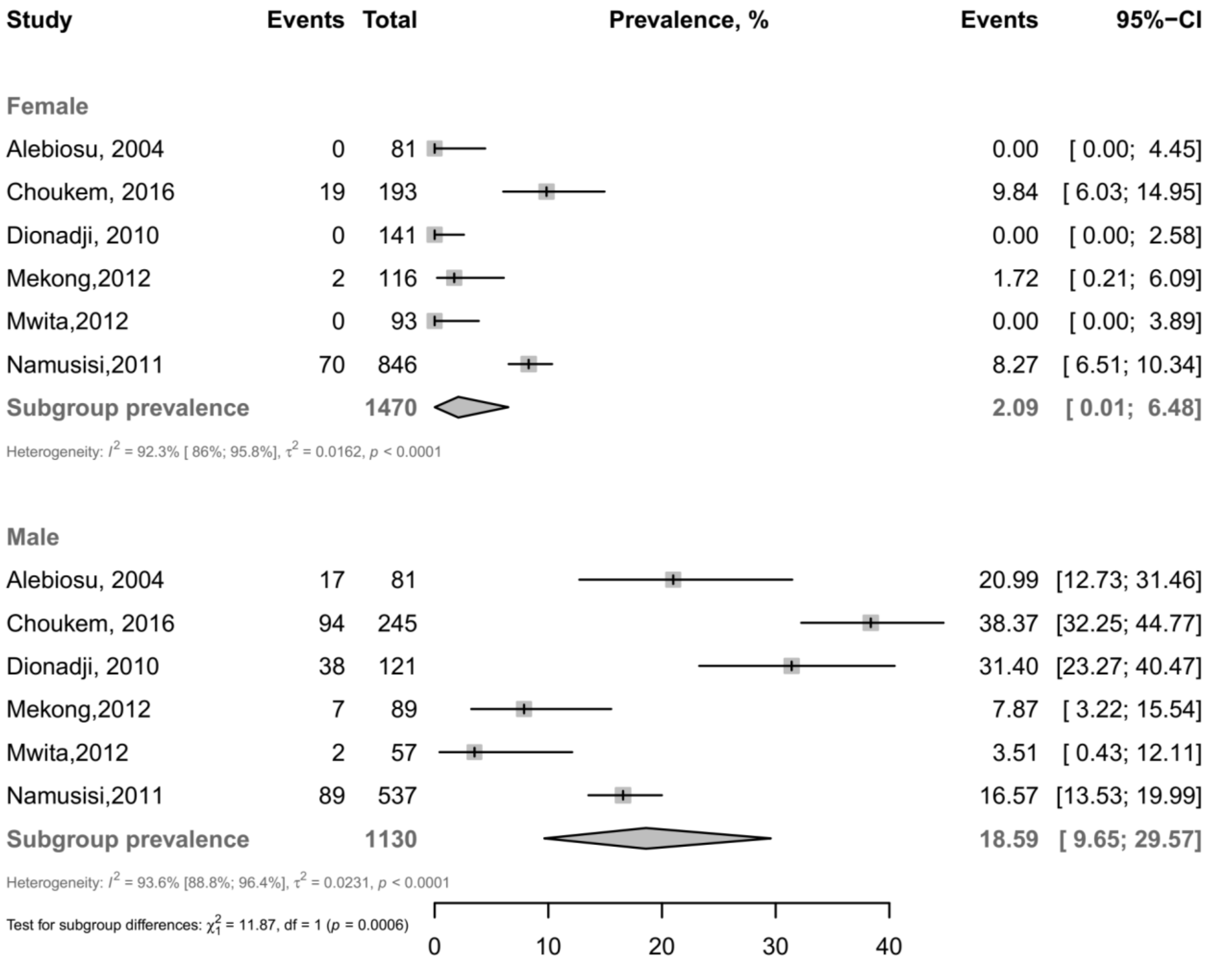

Supplementary Figure 7 : Prevalence of active smoking in people with diabetes mellitus in Africa according to sub-regions

Study	Events	Total	Events	95%−CI
**Central Africa**				
Choukem, 2007	47	210	22.38	[16.93; 28.63]
Choukem, 2016	202	438	46.12	[41.38; 50.91]
Jingi, 2016	2	134	1.49	[0.18; 5.29]
Mekong,2012	9	205	4.39	[2.03; 8.17]
**Subgroup prevalence**		**987**	**14.95**	**[0.93; 40.61]**
Heterogeneity: I^2 = 98.8% [98.1%; 99.2%], τ^2 = 0.0875, $p < 0.0001$				
**Eastern Africa**				
Ayah, 2013	7	66	10.61	[4.37; 20.64]
Dickson, 2016	336	701	47.93	[44.18; 51.70]
Gill, 2008	2	105	1.90	[0.23; 6.71]
Kirui,2012	53	1376	3.85	[2.90; 5.01]
Muhamedhussein,2016	0	58	0.00	[0.00; 6.16]
Mwita,2012	2	150	1.33	[0.16; 4.73]
Namusisi,2011	159	1383	11.50	[9.86; 13.30]
Ndege,2014	5	218	2.29	[0.75; 5.27]
Otieno, 2006	31	211	14.69	[10.21; 20.20]
Tamiru, 2010	14	256	5.47	[3.02; 9.01]
Wondemagegn, 2017	5	83	6.02	[1.98; 13.50]
**Subgroup prevalence**		**4607**	**7.31**	**[1.81; 15.81]**
Heterogeneity: I^2 = 98.6% [98.2%; 98.9%], τ^2 = 0.0488, $p < 0.0001$				
**Northern Africa**				
Amrane, 2012	14	178	7.87	[4.37; 12.84]
Ben Ahmed, 2012	70	126	55.56	[46.44; 64.40]
Bentata, 2016	81	671	12.07	[9.70; 14.78]
Bouzid, 2011	34	120	28.33	[20.49; 37.28]
Dinar, 2015	53	240	22.08	[17.00; 27.87]
Dionadji, 2010	38	262	14.50	[10.47; 19.36]
El−Shazly, 2002	268	1000	26.80	[24.08; 29.66]
El Achhab, 2008	6	89	6.74	[2.51; 14.10]
Fatouh, 2009	37	384	9.64	[6.88; 13.04]
Fawzy, 2014	42	100	42.00	[32.20; 52.29]
Khalil, 2016	22	80	27.50	[18.10; 38.62]
**Subgroup prevalence**		**3250**	**21.30**	**[14.50; 29.00]**
Heterogeneity: I^2 = 95.7% [93.9%; 97%], τ^2 = 0.0207, $p < 0.0001$				
**Southern Africa**				
Adeniyi, 2015	51	357	14.29	[10.83; 18.35]
Distiller, 2006	23	148	15.54	[10.11; 22.40]
Dunbar, 2015	258	593	43.51	[39.47; 47.61]
Makinga,2013	28	189	14.81	[10.08; 20.69]
Mengesha,2006	16	401	3.99	[2.30; 6.40]
Thomas, 2013	909	5515	16.48	[15.51; 17.49]
**Subgroup prevalence**		**7203**	**16.79**	**[8.38; 27.33]**
Heterogeneity: I^2 = 98.3% [97.5%; 98.8%], τ^2 = 0.0246, $p < 0.0001$				
**Western Africa**				
Abidoye, 2002	1	33	3.03	[0.08; 15.76]
Agunloye, 2013	73	400	18.25	[14.59; 22.39]
Alebiosu, 2004	17	162	10.49	[6.23; 16.27]
Bello−Sani, 2009	4	150	2.67	[0.73; 6.69]
Egbi, 2015	2	24	8.33	[1.03; 27.00]
Mbaye,2011	3	148	2.03	[0.42; 5.81]
Ogedengbe, 2014	16	124	12.90	[7.56; 20.11]
Oguntibeju, 2012	9	100	9.00	[4.20; 16.40]
**Subgroup prevalence**		**1141**	**7.72**	**[3.46; 13.32]**
Heterogeneity: I^2 = 87.6% [77.8%; 93.1%], τ^2 = 0.0135, $p < 0.0001$				

Test for subgroup differences: χ^2_4 = 11.56, df = 4 (p = 0.0209)

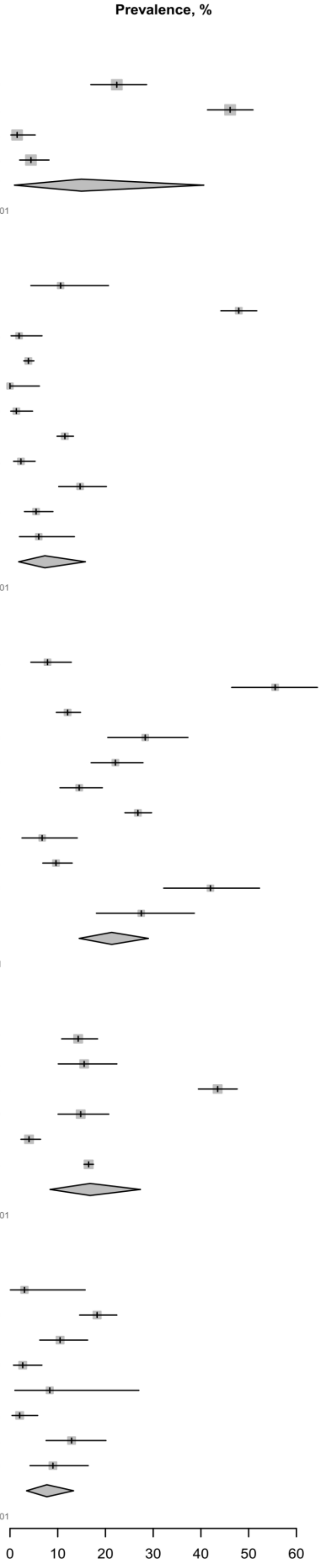

Supplementary Figure 8 : Prevalence of active smoking in people with diabetes mellitus in Africa according to area

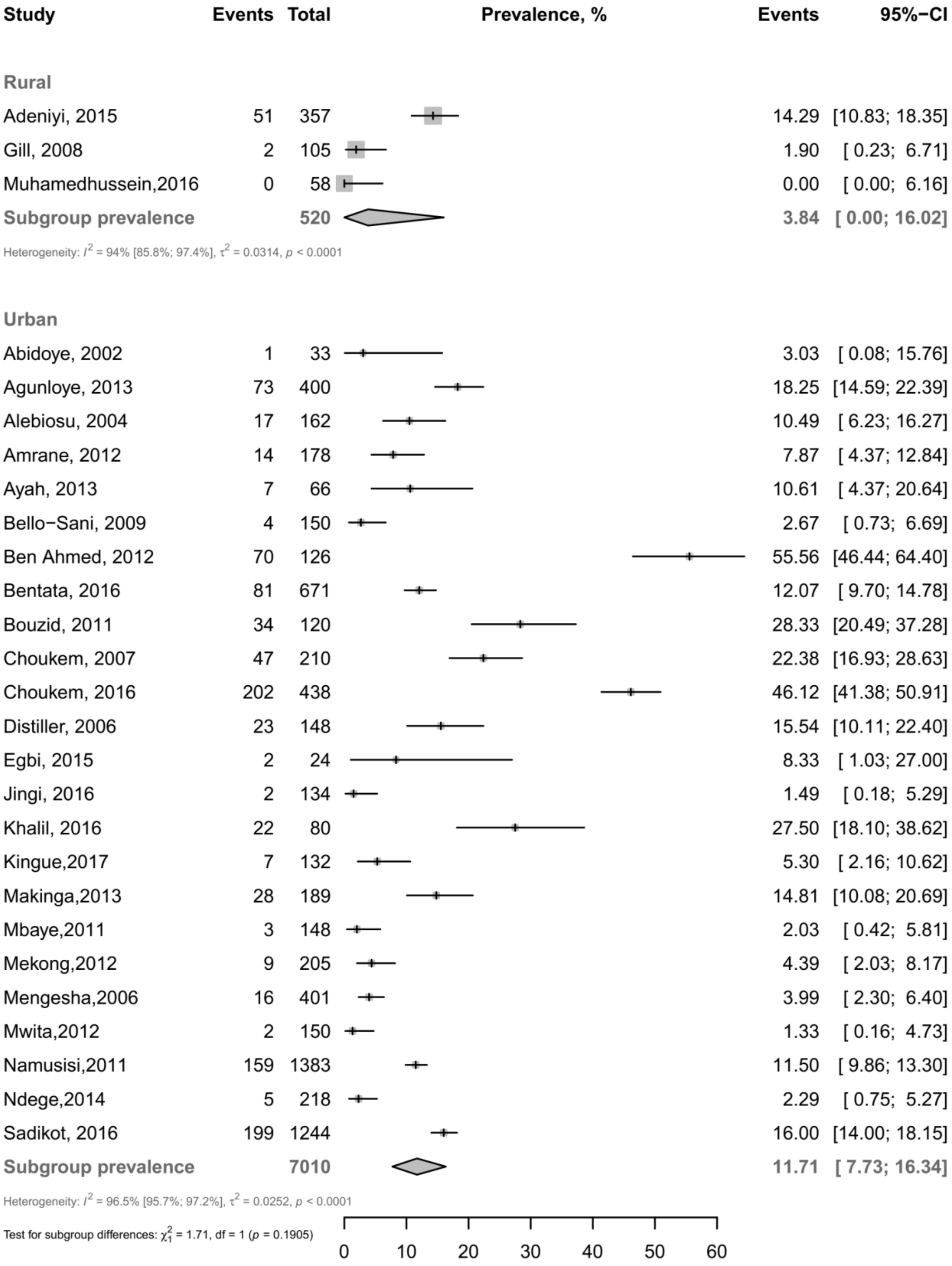

Supplementary Figure 9 : Prevalence of active smoking in people with diabetes mellitus in Africa according to setting

Study	Events	Total	Events	95%−CI
**Hospital**				
Agunloye, 2013	73	400	18.25	[14.59; 22.39]
Alebiosu, 2004	17	162	10.49	[6.23; 16.27]
Amrane, 2012	14	178	7.87	[4.37; 12.84]
Bello−Sani, 2009	4	150	2.67	[0.73; 6.69]
Ben Ahmed, 2012	70	126	55.56	[46.44; 64.40]
Bentata, 2016	81	671	12.07	[9.70; 14.78]
Bouzid, 2011	34	120	28.33	[20.49; 37.28]
Choukem, 2007	47	210	22.38	[16.93; 28.63]
Choukem, 2016	202	438	46.12	[41.38; 50.91]
Dickson, 2016	336	701	47.93	[44.18; 51.70]
Dinar, 2015	53	240	22.08	[17.00; 27.87]
Dionadji, 2010	38	262	14.50	[10.47; 19.36]
Distiller, 2006	23	148	15.54	[10.11; 22.40]
Dunbar, 2015	258	593	43.51	[39.47; 47.61]
Egbi, 2015	2	24	8.33	[1.03; 27.00]
El−Shazly, 2002	268	1000	26.80	[24.08; 29.66]
El Achhab, 2008	6	89	6.74	[2.51; 14.10]
Fatouh, 2009	37	384	9.64	[6.88; 13.04]
Fawzy, 2014	42	100	42.00	[32.20; 52.29]
Gill, 2008	2	105	1.90	[0.23; 6.71]
Jingi, 2016	2	134	1.49	[0.18; 5.29]
Khalil, 2016	22	80	27.50	[18.10; 38.62]
Kingue,2017	7	132	5.30	[2.16; 10.62]
Kirui,2012	53	1376	3.85	[2.90; 5.01]
Makinga,2013	28	189	14.81	[10.08; 20.69]
Mekong,2012	9	205	4.39	[2.03; 8.17]
Mengesha,2006	16	401	3.99	[2.30; 6.40]
Mwita,2012	2	150	1.33	[0.16; 4.73]
Namusisi,2011	159	1383	11.50	[9.86; 13.30]
Ndege,2014	5	218	2.29	[0.75; 5.27]
Ogedengbe, 2014	16	124	12.90	[7.56; 20.11]
Oguntibeju, 2012	9	100	9.00	[4.20; 16.40]
Otieno, 2006	31	211	14.69	[10.21; 20.20]
Tamiru, 2010	14	256	5.47	[3.02; 9.01]
Thomas, 2013	909	5515	16.48	[15.51; 17.49]
**Subgroup prevalence**		**16575**	**14.29**	**[10.45; 18.60]**
**Population**				
Abidoye, 2002	1	33	3.03	[0.08; 15.76]
Adeniyi, 2015	51	357	14.29	[10.83; 18.35]
Ayah, 2013	7	66	10.61	[4.37; 20.64]
Mbaye,2011	3	148	2.03	[0.42; 5.81]
Muhamedhussein,2016	0	58	0.00	[0.00; 6.16]
Sadikot, 2016	199	1244	16.00	[14.00; 18.15]
Wondemagegn, 2017	5	83	6.02	[1.98; 13.50]
**Subgroup prevalence**		**1989**	**6.61**	**[2.53; 12.23]**

Hospital — Heterogeneity: I^2 = 98% [97.6%; 98.3%], τ^2 = 0.0288, p = 0

Population — Heterogeneity: I^2 = 91% [84%; 94.9%], τ^2 = 0.0134, p < 0.0001

Test for subgroup differences: χ^2_1 = 4.61, df = 1 (p = 0.0319)

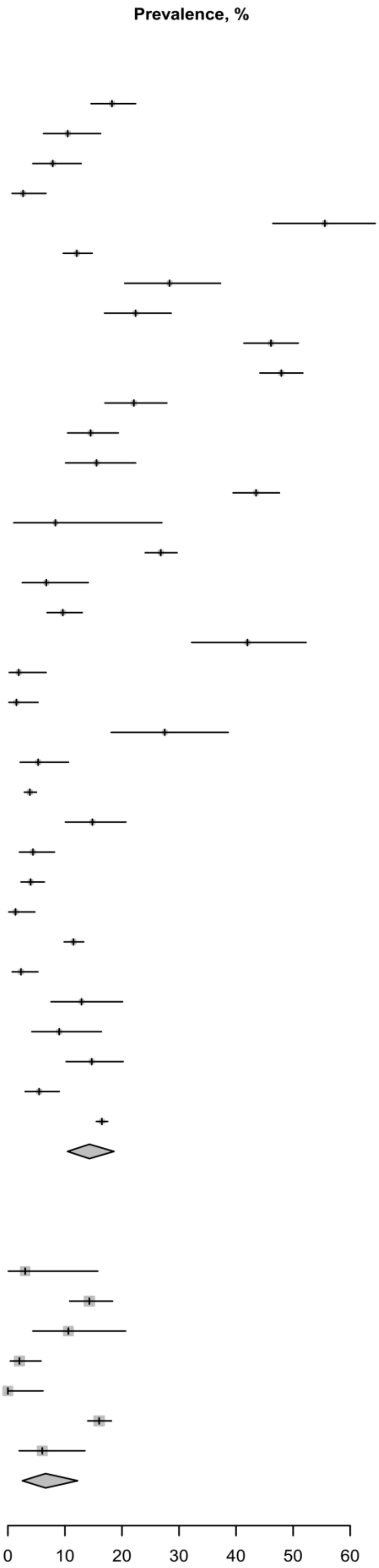

Supplementary Figure 10 : Funnel plot for publication bias in the prevalence of active smoking in people with hypertension in Africa

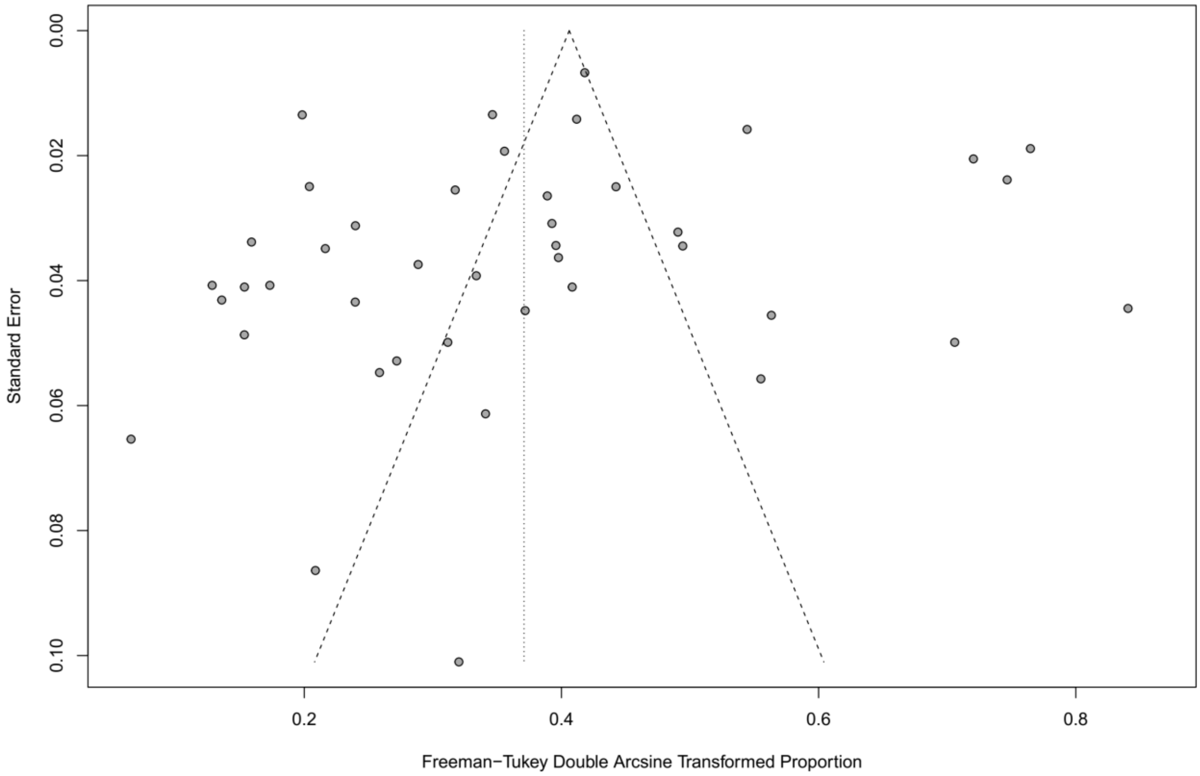

Supplement: Supplementary file 1 — Supplementary Information [file 41598_2018_37858_MOESM1_ESM.pdf]
